# A novel method based on high‐resolution imaging spectroscopy to predict fine‐root traits and the economics space of fresh tree roots

**DOI:** 10.1111/nph.71366

**Published:** 2026-06-23

**Authors:** Naoki Makita, Natsuko Tanikawa, Tatsuro Nakaji

**Affiliations:** ^1^ Faculty of Science Shinshu University Nagano 390‐8621 Japan; ^2^ Sapporo Experimental Forest Hokkaido University Sapporo 060‐0809 Hokkaido Japan

**Keywords:** chemical composition, fresh roots, image processing, nondestructive analysis, root economics space, root structure, spectral reflectance

## Abstract

- Tree fine‐root morphological, anatomical, and chemical traits are important to reflect belowground resource acquisition strategies to support tree growth, but their measurements involve several labor‐intensive and time‐consuming procedures. We examined the efficacy of visible (VIS), near‐infrared (NIR), and shortwave infrared region (SWIR) spectral reflectance for predicting a comprehensive set of fine‐root traits.
- Spectral features of fresh fine roots in 20 tree species were analyzed using three distinct spectral datasets: the VIS–NIR (458–960 nm), the SWIR (967–2391 nm), and the combined spectral region (full‐range; 458–2391 nm). The partial least square regression (PLSR) model using root spectral reflectance was developed to estimate nine functional root traits.
- Comparison of the models revealed that the prediction accuracy was higher when using the SWIR and full‐range datasets than when using the VIS–NIR dataset. The trait with the highest prediction accuracy was root diameter. The predicted multiple root traits successfully reproduced the ordination of the multidimensional trait space, exhibiting a pattern highly similar to that of the observed traits.
- This method can advance our understanding of multidimensional fine‐root trait spaces and resource strategies by enabling efficient measurement and spatial modeling of root traits across diameter classes.

## Graphical abstract

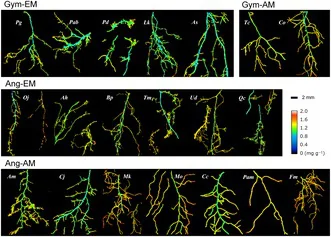

See also the Commentary on this article by Rewald, **252**: 1695–1699.

## Introduction

Tree fine roots, generally defined as roots having ≤ 2.0 mm in diameter, play important roles in carbon, water, and nutrient cycling (Bardgett *et al*., 2014; Ma *et al*., 2018). Although fine roots constitute only a small part of the total root biomass, they contribute to the function of fine roots in resource acquisition and to the large fractions of the annual net primary production, soil CO_2_ fluxes, and carbon accumulation in forest soils (Finér *et al*., 2011; Makita *et al*., 2011; Ma *et al*., 2018). The morphological, chemical, and physiological traits of fine roots are correlated with their roles in various plant functional processes (Weemstra *et al*., 2016; McCormack *et al*., 2017). For instance, a positive correlation between specific root length (SRL) and root respiration and exudation rates is widely recognized, interpreted as reflecting the high metabolic costs associated with active nutrient acquisition and tissue maintenance in roots with high SRL (Makita *et al*., 2009; Makita *et al*., 2016; Akatsuki & Makita, 2020). Furthermore, despite the significant ecological importance of root traits, this field of research is constrained by the substantial effort required to measure the root traits.

There is growing evidence that fine‐root traits are important for understanding plant resource acquisition and conservation strategies (Carmona *et al*., 2021). Recent advancements have shifted the conceptual framework from a one‐dimensional ‘root economics spectrum’ to a multidimensional ‘root economics space’ (RES) (Bergmann *et al*., 2020; Sanaei *et al*., 2026). This framework posits that variation is driven by two orthogonal axes: conservation and collaboration. The conservation axis represents a trade‐off between resource acquisition (e.g. nitrogen (N) concentration) and tissue persistence (e.g. root tissue density (RTD), cellulose, and lignin). Meanwhile, the collaboration axis describes the trade‐off between independent soil exploration (‘do‐it‐yourself’; e.g. high SRL) and reliance on mycorrhizal fungi (‘outsourcing’; e.g. thicker roots and higher cortex ratios). Despite this conceptual clarity, understanding trait coordination remains difficult because methodological limitations have hindered detailed mapping of this economics space. Traditional analyses require substantial biomass, necessitating sample pooling and grinding, thereby obscuring natural variability and precluding the study of small, intact samples.

In the past decades, spectroscopy has been used for measuring and understanding the traits of plant organs (Foley *et al*., 1998; Asner *et al*., 2011; Rewald & Meinen, 2013; Serbin *et al*., 2014; Nakaji *et al*., 2019; Burnett *et al*., 2021) and soil properties (Bellon‐Maurel & McBratney, 2011; Knox *et al*., 2015; Nakaji *et al*., 2023). Spectroscopy utilizes the spectral reflectance or absorbance of the target sample in the VIS (350–700 nm), NIR (700–1100 nm), SWIR (1100–2500 nm), and/or longer spectral wavelength at mid‐infrared (2.5–25 μm) spectral range. Although waveband designations vary by field – for instance, NIR often includes SWIR in chemical analysis – this study focuses on the VIS, NIR, and SWIR (458–2391 nm) regions where reflectance features relate to physical and chemical characteristics, such as C–H, N–H, and O–H bond absorption in the SWIR range (Osborne *et al*., 1993; Ciurczak, 2001). Combining spectral data with modeling such as partial least square regression (PLSR) or machine learning enables rapid estimation of organic components (Chang *et al*., 2001; Ludwig & Khanna, 2001; Tong *et al*., 2010; Knox *et al*., 2015). Imaging spectroscopy is particularly advantageous for measuring small samples such as root orders and evaluating spatial trait distributions. While these methods are well‐established for leaf traits and standardized for remote sensing (Asner *et al*., 2011; Rewald & Meinen, 2013; Serbin *et al*., 2014; Burnett *et al*., 2021; Nakaji *et al*., 2023), their application to fresh, living fine roots remains a critical frontier to alleviate the labor‐intensive nature of traditional measurements.

Spectroscopic analysis has been applied in root ecology to quantify live/dead roots (Roumet *et al*., 2006; Rewald & Meinen, 2013; Domisch *et al*., 2015), identify species (Lei & Bauhus, 2010), and predict C, N, and lignin concentrations (Picon‐Cochard *et al*., 2009; Elle *et al*., 2019). However, most applications have relied on dried, ground samples to ensure stability and accuracy, a process that destroys critical structural features. Because studies on fresh, living roots remain scarce (Nakaji *et al*., 2008; Bodner *et al*., 2018; Tanikawa *et al*., 2019), it is still unclear how imaging spectroscopy can reveal functional traits and their spatial distribution across root diameter classes. Since fine‐root research is currently hindered by time‐consuming traditional measurements compared with leaf studies, the use of spectral data from intact samples could alleviate this bottleneck by providing a more efficient means of analysis.

To determine the relationship between the spectral reflectance of fresh roots and fine‐root traits, Tanikawa *et al*. (2019) analyzed the pattern and magnitude of VIS–NIR spectral reflectance at 427–977 nm of intact and fresh fine‐root systems using a spectral imager. Although they reported significant relationships between spectral reflectance at specific wavelengths and functional root traits of 12 tree species, the spatial distribution of traits of the root systems has not yet been developed and evaluated. Furthermore, imaging spectroscopy can capture the spatial distribution of target traits based on spectral image data and visualize the characteristics of fresh fine roots without chemical processing or sampling at the fine‐scale level (Nakaji *et al*., 2008, 2019). This technique has the potential to address methodological limitations in root ecology (Rewald & Meinen, 2013), particularly in fine‐scale characterization of trait distributions across root orders and functional groups.

To address these methodological limitations, this study developed a high‐resolution imaging spectroscopy method for predicting multidimensional fine‐root traits from small, fresh, and intact samples. This approach enables examination of intact root segments without destructive processing, thereby providing deeper insights into trait coordination within the RES. We expected that imaging spectroscopy could predict fine‐root traits in a way that reproduces the ordination of the feature space similar to the observed features. To test this, first, the feasibility of imaging spectroscopy at the VIS–NIR and SWIR spectral regions for evaluating the fine‐root traits of intact and fresh samples was explored. Structural root traits such as SRL, RTD, and stele and cortex ratios, and chemical traits such as N, cellulose, and lignin concentrations, and C : N ratio of different tree species along the root diameter gradient were analyzed. To understand the similarities and differences among woody species, fine roots of 20 tree species with different types of phylogenies and mycorrhizal symbiosis were collected from a cool‐temperate forest in Japan. In addition, the main attributes of fine‐root traits on the RES were analyzed using each observed and predicted trait and compared. Specifically, we tested the hypotheses that (1) the model approaches using VIS–NIR and SWIR spectral reflectance are effective at predicting root traits, and (2) the coordination of multidimensional traits between observed and predicted traits is consistent. Finally, we applied our models to construct a two‐dimensional image of root trait distribution within the root system.

## Materials and Methods

### Study site

The root sampling was conducted in natural deciduous broad‐leaved forest and artificial coniferous forests in Tomakomai Experimental Forest, Hokkaido University, Japan (42°42′31″N, 141°33′56″E). The study area is located on flat land, and the mean annual precipitation and air temperature are 1112 mm and 6.7°C, respectively. Monthly mean air temperature ranges from −9.5°C to 22.2°C. The soil type can be classified as immature soils or Regosols according to the definitions of the forest soil division in Japan and international soil classification systems, respectively (Urakawa *et al*., 2015). The main parent material until *c*. 1.5 m depth originated from the volcanic ash deposited from the eruption of Mt. Tarumae in 1667. The depth of the organic soil layer is < 0.3 m, which has developed on a 2‐m‐deep volcanic soil layer. The mean soil electrical conductivity and pH are 115.0 μS cm^−1^ and 5.2, respectively. The dominant tree species in natural deciduous broad‐leaved forests are *Quercus crispula*, *Acer pictum*, *Sorbus alnifolia*, and *Tilia maximowicziana* (Hiura, 2001). In artificial coniferous forests, one deciduous species, *Larix kaempferi*, and more than 30 evergreen species, such as *Picea glehnii*, *Abies sachalinensis*, and *Chamaecyparis obtusa*, were planted for > 40 yr before this study. We collected fine‐root samples from 20 tree species (Table 1) that included two phylogenetic groups, gymnosperms and angiosperms, and two mycorrhizal symbiotic groups, ectomycorrhiza (EM) and arbuscular mycorrhizas (AM). Tree species were classified into four groups: gymnosperm–EM, gymnosperm–AM, angiosperm–EM, and angiosperm–AM (Smith & Read, 2008).

**Table 1:** Taxonomy, mycorrhizal symbiosis, and leaf habits of 20 tree species.

Species	Abbreviation	Family	Taxonomy	Symbiosis	Leaf habit
*Picea glehnii* (F.Schmidt) Mast.	*Pg*	Pinaceae	Gymnosperm	EM	Evergreen
*Picea abies* (L.) H.Karst.	*Pab*	Pinaceae	Gymnosperm	EM	Evergreen
*Pinus densiflora* Siebold et Zucc.	*Pd*	Pinaceae	Gymnosperm	EM	Evergreen
*Larix kaempferi* (Lamb.) Carrière	*Lk*	Pinaceae	Gymnosperm	EM	Deciduous
*Abies sachalinensis* (F.Schmidt) Mast.	*As*	Pinaceae	Gymnosperm	EM	Evergreen
*Taxus cuspidata* Siebold et Zucc.	*Tc*	Taxaceae	Gymnosperm	AM	Evergreen
*Chamaecyparis obtusa* (Siebold et Zucc.) Endl.	*Co*	Cupressaceae	Gymnosperm	AM	Evergreen
*Ostrya japonica* Sarg.	*Oj*	Coryloideae	Angiosperm	EM	Deciduous
*Alnus hirsuta* Turcz.	*Ah*	Betulaceae	Angiosperm	EM	Deciduous
*Betula platyphylla* Sukaczev	*Bp*	Betulaceae	Angiosperm	EM	Deciduous
*Tilia maximowicziana* Shiras.	*Tm*	Tiliaceae	Angiosperm	EM	Deciduous
*Ulmus davidiana* var. japonica	*Ud*	Ulmaceae	Angiosperm	EM	Deciduous
*Quercus crispula* Blume var. crispula	*Qc*	Fagaceae	Angiosperm	EM	Deciduous
*Acer mono* Maxim.	*Am*	Sapindaceae	Angiosperm	AM	Deciduous
*Cercidiphyllum japonicum* Sieb. & Zucc.	*Cj*	Cercidiphyllaceae	Angiosperm	AM	Deciduous
*Magnolia kobus* DC. var. borealis Sarg.	*Mk*	Magnoliaceae	Angiosperm	AM	Deciduous
*Magnolia obovata* Thunb.	*Mo*	Magnoliaceae	Angiosperm	AM	Deciduous
*Cornus controversa* Hemsl. var. controversa	*Cc*	Cornaceae	Angiosperm	AM	Deciduous
*Phellodendron amurense* Rupr. var. amurense	*Pam*	Rutaceae	Angiosperm	AM	Deciduous
*Fraxinus mandshurica* Rupr.	*Fm*	Oleaceae	Angiosperm	AM	Deciduous

AM, arbuscular mycorrhizas; EM, ectomycorrhiza.

### Root sampling

Fine‐root samples of all 20 species were collected over 3 wk, from mid‐August to early September, in both 2017 and 2018. In each year, three trees per species were sampled, resulting in a total of six trees per species. The diameter at breast height of the targeted trees was 27.3 ± 6.8 cm (mean ± SD). Large root segments around a target tree were located and traced back to the parent trunk to identify the tree species within a 3 m radius surrounding the trunk. The intact root systems of each target tree were gently exposed from the soil and excised using pruning shears and small knives. Two samples were collected from the same individual, close to each other, and one sample was used for reflectance measurements, morphological, and chemical traits, while the other was used to assess anatomical traits. All the collected segments were placed in a cooler to retain moisture and transported to the laboratory within 1 h.

In the laboratory, the root segments were gently washed with tap and distilled water to remove the attached soil. The segments of living fine roots (≤ 2.0 mm in diameter) were selected based on root color and structure. Fine roots with a light color and intact stele and periderm are usually regarded as living roots (Vogt & Persson, 1991). The living intact root systems were further classified into three categories based on the thickness of the main fine‐root axis; diameter < 0.5, 0.5–1.0, and 1.0–2.0 mm. A total of 360 (20 species × 6 replicates × 3 classes based on diameter) root samples were analyzed. In this study, we examined the spectral reflectance, morphology, chemistry, and anatomy of fine roots.

### Spectral reflectance measurement

Immediately after sampling, we started to measure the spectral reflectance imaging of root segments. The hyperspectral reflectance images of the root segments were obtained using two line‐scan spectral camera systems with sensitivity in the VIS–NIR and SWIR spectral regions (Table 2). A system similar to that reported by Tanikawa *et al*. (2019) was used for VIS–NIR imaging after modification in the range of the analyzed spectral range. The maximum range of the analyzed spectral region obtained by combining two camera systems was 458.4–2390.8 nm, which was captured as 370 bands at 4.11‐ to 5.79‐nm scanning intervals (Table 2). The data at wavelengths less than 458.4 nm were excluded from analysis due to the low signal : noise ratio, which was < 25 (i.e. > 4% noise). The data at the overlapping spectral regions of the two camera systems were omitted before analysis.

**Table 2:** Characteristics of hyperspectral reflectance imaging systems, named visible–near‐infrared (VIS–NIR) and shortwave infrared (SWIR).

	VIS–NIR	SWIR
Spectral range (nm)	458.4–959.6	966.7–2390.8
Sampling interval (nm)	4.11	5.79
Number of bands	123	247
FWHM (nm)	5–10	9.1
Spatial resolution (mm)	0.1	0.1
Lens	Cinegon 1.4/12 (Joe. Schneider Optische Werke GmbH., Bad Kreuznach, Germany)	TFO‐30 (Specim Spectral Imaging Ltd, Oulu, Finland) with 7‐mm spacer
Imaging spectrograph	ImSpector V10 (Specim Spectral Imaging Ltd., Oulu, Finland)	ImSpector N24E (Specim Spectral Imaging Ltd, Oulu, Finland)

Fresh root samples were wrapped with wet tissue paper and stored at cool temperatures until imaging was performed to retain their moisture. Each fine‐root sample was arranged on a low‐reflectance background plate (black cloth; spectral reflectance < 4%). The sample plate was placed on a linear motorized stage set below the imaging system (Fig. 1a), wherein one shot of the spectral camera image corresponds to the vertical linear area of the field of view. Spectral intensity images were successively captured by moving the sample plate horizontally and connecting the continuous data to generate a two‐dimensional image. We carried out this process with both the VIS–NIR and SWIR imaging systems. Since the two systems could not be activated simultaneously, SWIR imaging was performed the day after the VIS–NIR. Because the root samples were rearranged during SWIR imaging, the roots' positions on the plate were slightly different between NIR and SWIR. For this reason, in the subsequent image analysis, we calculated the root spectral reflectance separately in each spectral region without combining the VIS–NIR and SWIR images. These spectral datasets were then merged using a weighted averaging technique at the 960‐ to 967‐nm junction to eliminate offsets, ensuring a continuous spectral curve (458–2391 nm) for subsequent PLSR and principal component analysis.

**Fig. 1:**
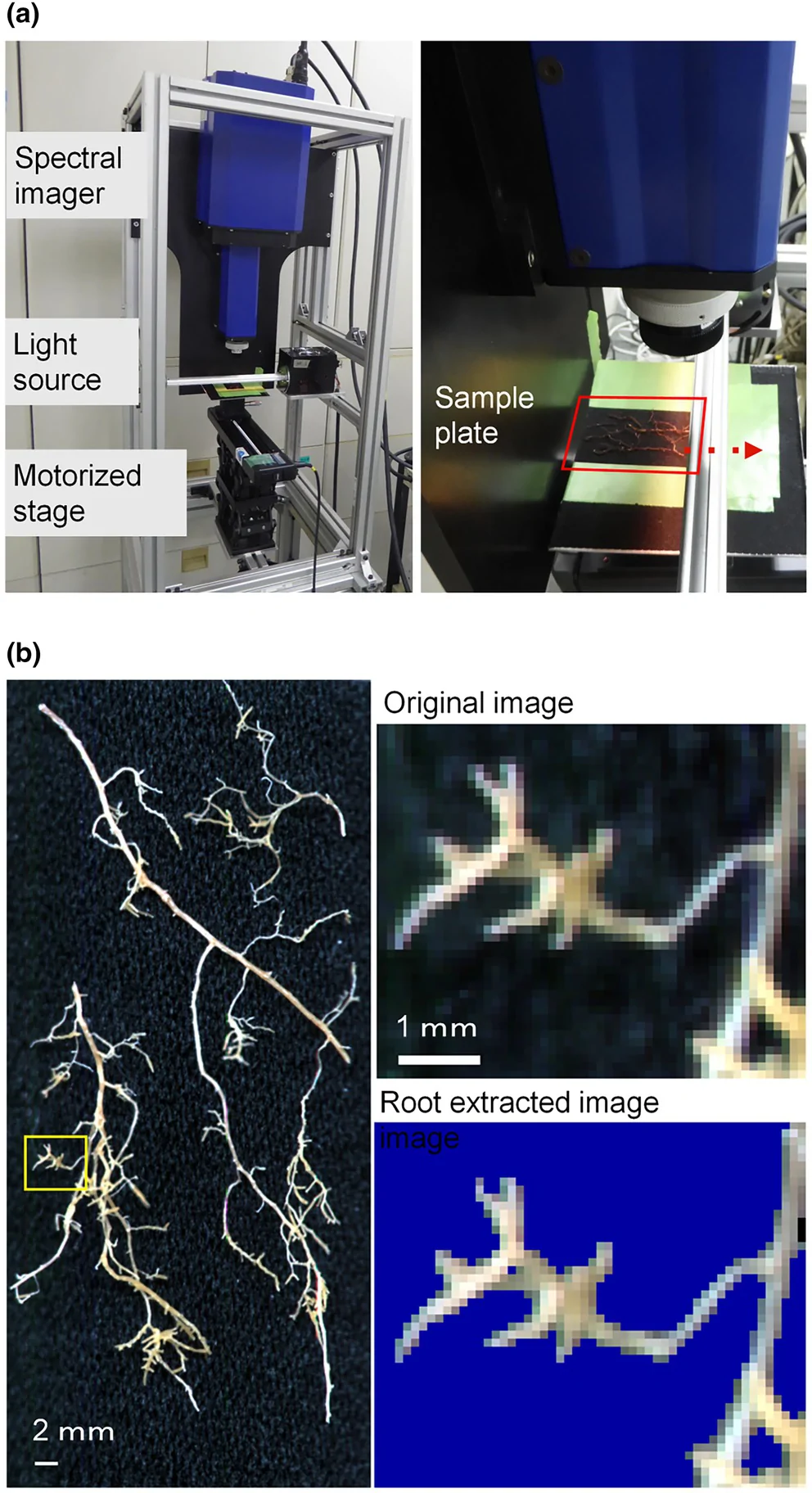
Schematic representation of the hyperspectral imaging system and the pixel selection process for fine roots. (a) Photograph of shortwave infrared (SWIR) hyperspectral camera system and (b) sample images of SWIR reflectance of *Quercus crispula*. Fine roots of the same class and the same individual are photographed in one image. The sample plate indicated by a red square in (a) slides horizontally during image capture. The SWIR camera is shown as a representative example; the visible–near‐infrared (VIS–NIR) system utilizes a virtually identical configuration, including the lighting system and sample stage. The two images on the right in (b) are enlarged views of the yellow‐squared area and the result of root extraction (bottom right). Pixels of the background parts shown as a blue‐colored area are removed when the mean reflectance of the sample roots is calculated.

In both imaging systems, the spatial resolution of the line image and the speed of the horizontal movement were adjusted to 0.1 mm per pixel and 0.1 mm per shot, respectively, resulting in 0.1‐mm spatial resolution at both the vertical and horizontal directions (Fig. 1b). A halogen lamp and a diffuse reflectance plate (Spectralon; Labsphere Inc., North Sutton, NH, USA) were used as the irradiation light source and reflectance standard, respectively. Since the reflectance of the root organ is generally < 50%, we used a 50% diffuse reflectance plate as a standard to calculate the spectral reflectance of the target root sample. This selection was made to optimize the sensor's dynamic range and improve the signal‐to‐noise ratio by more closely matching the reflectance levels of the target samples to those of the reference standard, a common practice in proximal spectroscopy of dark biological tissues (Smith & Milton, 1999). All images were captured within 3 d after root sampling.

### Image analysis

The mean spectral reflectance of the root sample was calculated using the extracted root pixel values in each sample image. As the spectral reflectance features between the roots and background plate used in this study were different (Supporting Information Fig. S1), the image pixels were able to be separated into roots and backgrounds based on the spectral reflectance value in a specific band and the normalized difference index calculated using the two bands. In each of the VIS–NIR and SWIR images, the following empirical equations were used for separation (i.e. extraction of the root part). In a VIS–NIR reflectance image, a pixel is discriminated as a root when

(Eqn 1) $$R_{889.7}\geq 9.32\;\text{and}\;\frac{R_{635.0}-R_{478.9}}{R_{635.0}+R_{478.9}}\geq 0.23$$

or

(Eqn 2) $$R_{889.7}\geq 9.32\;\text{and}\;\frac{R_{836.3}-R_{462.5}}{R_{836.3}+R_{462.5}}\geq 0.23$$

In SWIR reflectance image, a pixel was discriminated as root when

(Eqn 3) $$R_{1609.3}\geq 8.96\;\text{and}\;\frac{R_{1290.9}-R_{1974.0}}{R_{1290.9}+R_{1974.0}}\geq 0.20$$

In the equations mentioned previously, *R* and the following numbers indicate the spectral reflectance (%) and wavelength (nm), respectively. To isolate root pixels from the background, we used spectral thresholding, a key technique in remote sensing for image segmentation based on reflectance contrast at specific wavelengths. Two thresholds, as shown in Eqns 1 and 2, were used to differentiate root surface reflectance among species. Specifically, different thresholds were necessary for three species (2 *Magnolia* and *Phellodendron*) because their spectral signatures varied significantly from the other 17 species. All threshold values and wavelengths used were determined based on the comparison between the manually extracted pixel values of the background and roots using a portion of the image data in the preliminary analysis (Fig. S1). In a preliminary analysis, we searched for indices common to all species; however, for the three species belonging to angiosperms and AM, using differences in reflectance within the VIS region (479 and 635 nm) provided higher separability than using VIS and NIR wavelengths (which were common to all 17 species). Therefore, different wavelengths and thresholds were used for these three species. Image processing was conducted using the Erdas Imagine software (Leica Geosystems Geospatial Imaging, GA, USA). In calibration of the trait estimation model based on spectral reflectance described below, the mean root reflectance within the image was used.

### Measurements of root morphology and chemistry

In parallel with the works mentioned previously, digital grayscale images of the fresh root segments were obtained using a double‐lamp bed scanner (GT‐X980; Epson, Nagano, Japan) for analyzing the morphological root traits. The total length and volume of the root segments were estimated using the scanned image with the WinRHIZO Pro 2013a software (Regent Instruments, Quebec, QC, Canada). The samples were then dried at 70°C to a constant weight to determine the root mass. The SRL (m g^−1^) and RTD (g cm^−3^) of the total root length, volume, and dry mass of the samples were calculated following the Freschet *et al*. (2021). Then, the dried root samples were ground to fine powder using a TissueLyser (Qiagen) for chemical analysis. The N and C concentrations of the samples were quantified using an elemental analyzer (Flash 2000 Thermo Fisher Scientific Inc., Waltham, MA, USA). Lignin and cellulose concentrations were analyzed according to the methods described by Nakaji *et al*. (2019). For chemical traits, N concentration and C : N ratio were measured for all six individuals per species (*n* = 6). However, due to the large amount of biomass required for analysis, lignin and cellulose concentrations were determined using only the three individuals collected in 2018 (*n* = 3). It should be noted that lignin and cellulose analysis was conducted using the three root systems collected from different individuals in 2018, because the amounts of the sample required for this analysis were insufficient in the individuals sampled in 2017. In total, six individuals per species (three from 2017 and three from 2018) were used for other trait analyses, but only the 2018 subsets were utilized for these specific chemical components.

### Root anatomical measurements

The diameters of the stele and cortex for each branching order of the root system were measured for three individuals per species (*n* = 3), randomly selected from the six individuals used for morphological analysis. A freezing microtome (Leica CM1950; Leica, Tokyo, Japan) was used to cut the segments into 20‐μm‐thick cross sections. All the root cross sections were stained with safranin‐fast green, dehydrated in a graduated series (50, 70, 90, 95, and 100%) of ethanol, and then photographed under a polarizing microscope (BX51; Olympus, Tokyo, Japan). The root and stele diameters (mm) and cortex thickness (mm) were measured from one root cross section per diameter class. The root cross sections were subjected to anatomical measurements, following the method described by Endo *et al*. (2021).

### Estimation model using hyperspectral reflectance data

The PLSR was employed to develop a root characteristic prediction model using spectral reflectance data. The size of data points used for this analysis differed according to the target characteristics. The number of measurements for morphology, N concentration, and C : N ratio was 360 (20 species × 3 root classes × 6 individuals), while those for secondary metabolites (lignin and cellulose) and anatomical parameters were 180 (20 species × 3 root classes × 3 individuals). In the model analysis, the data were split into two‐thirds and one‐third to be used for the model training (calibration) and out‐of‐sample validation, respectively. The data splitting was conducted with the intention of avoiding the differences in species composition and root class between the training and validation datasets. Particularly, in the case of 360 data, the data of the randomly selected four individuals were used for calibration, and the remaining two individuals for validation. The differences between the training dataset and the validation dataset were statistically tested before the model analysis. The explanatory variable was centered before the regression to reduce multicollinearity, and other preprocessing steps for reflectance – such as calculating the derivative reflectance and applying log transformations – were not performed in this study due to minimal improvements in estimation accuracy. The best‐performing model for each trait was selected by comparing validation statistics (*r* ^2^, rRMSE, and RPD) across the three spectral regions.

(Eqn 4) $$\text{RMSE}={\left[\Sigma \;{\left(\text{estimated}-\text{measured}\right)}^{2}\right]}^{0.5}$$

(Eqn 5) $$\mathrm{RPD}=\frac{\mathrm{SD}}{\mathrm{SEP}}\;\text{or}\;\frac{\mathrm{SD}}{\text{SECV}}$$

where SD, SEP, and SECV are the SD of the training data, the SE of estimation during the calibration process, and the SE of cross‐validation, respectively. In addition, the spectral features of correlation coefficient, variable influence on projection (VIP; Wold *et al*., 2001) were calculated for understanding the importance of a specific wavelength in the PLSR model.

The performance of the three PLSR models was evaluated by using three distinct spectral datasets: the VIS–NIR dataset (458–960 nm), the SWIR dataset (967–2391 nm), and the combined spectral region (full‐range dataset; 458–2391 nm). These analyses of PLSR model calibration and independent validation were conducted using the statistical software XLSTAT Base (Lumivero, Denver, CO, USA).

### Statistical analysis

To evaluate variation in functional traits across biological groups and sizes, the traits of the three root classes based on diameter were subjected to a two‐way analysis of variance (ANOVA), with taxonomy (gymnosperm vs angiosperm) and mycorrhizal symbiosis (EM vs AM) as the main factors for each variable. Statistical analyses were performed using the XLSTAT Base.

To characterize the RES and assess the degree of similarity between the trait spaces derived from observed and predicted values, PCA was performed on morphological and chemical traits. The PCA was based on a correlation matrix of four key parameters (diameter, SRL, RTD, and N concentration) using the statistical package R 3.6.3 (R Development Core Team, 2020).

## Results

### Root spectral reflectance in 20 woody species

The spectral feature of root reflectance of the 20 tree species showed similar shapes in the phylogenetic and mycorrhizal symbiotic groups, regardless of diameter (Fig. 2; Dataset S1). In the VIS spectral region < 700 nm of wavelength, white color roots exhibited high reflectance. The reflectance increased sharply from the VIS to the NIR spectral region up to 900 nm for all the woody species (Fig. 2). In the SWIR region (967–2391 nm), the overall reflectance magnitude was lower than in the NIR region, primarily due to water absorption. Subsequently, reflectance was attenuated by absorption by H_2_O in the small valleys (970 and 1200 nm) and two large valleys (1450 and 1940 nm). Small troughs in the reflectance curve were observed around the absorption bands of cellulose and lignin (1780–1830 nm), and protein and N (2200 nm; Fig. 2). The reflectance magnitude also varied significantly among diameter classes and species. Generally, thinner roots (< 0.5 mm) exhibited higher reflectance across both VIS–NIR and SWIR regions compared with thicker roots (1.0–2.0 mm), likely reflecting lower tissue density. However, this diameter effect was group‐specific; for instance, thinner roots exhibited higher reflectance in the gym‐AM groups but relatively lower reflectance in the ang‐EM groups (Fig. 2a,b).

**Fig. 2:**
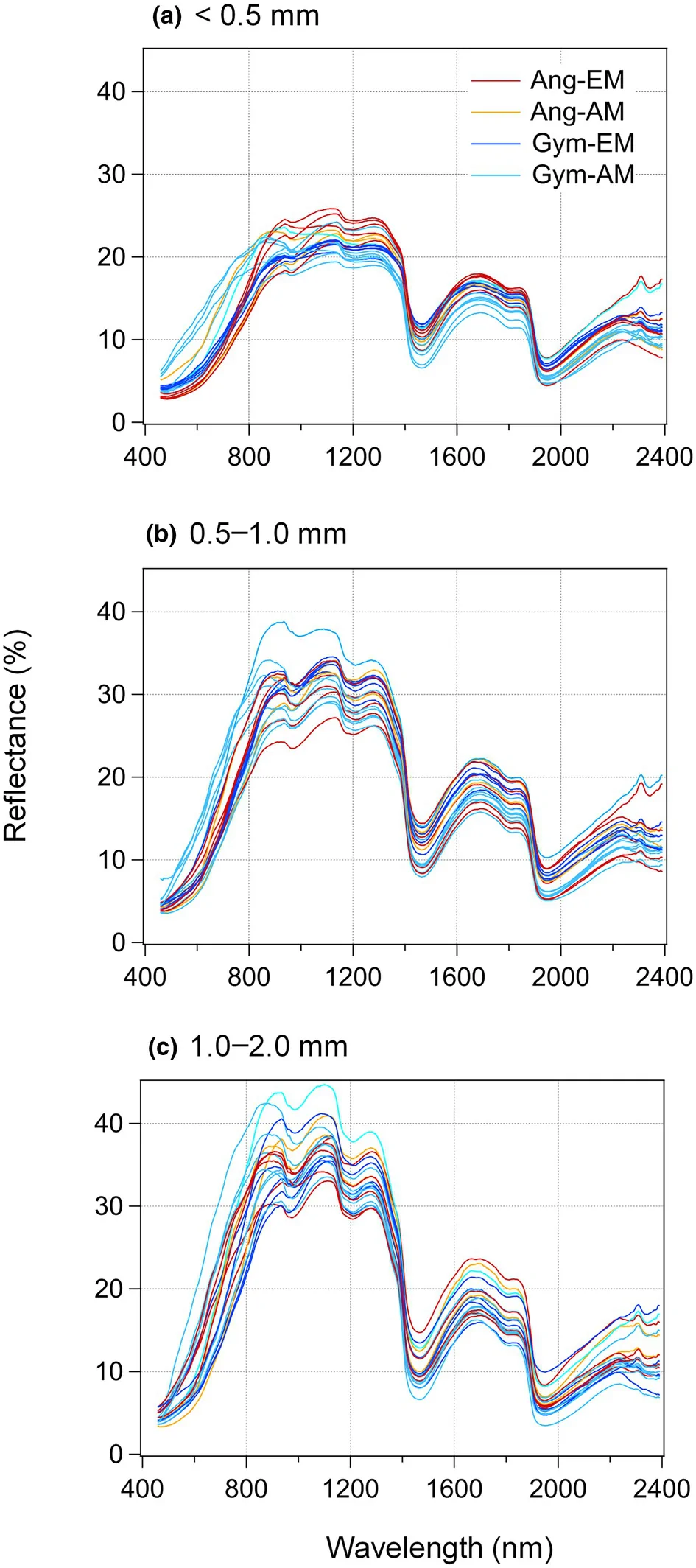
Spectral reflectance features of fresh roots of 20 tree species in three root classes based on diameter: (a) < 0.5 mm, (b) 0.5–1.0 mm, and (c) 1.0–2.0 mm. The reflectance values were averaged in each species (*n* = 6).

### Root traits in 20 woody species

Figs 3 and 4 represent the root morphological and chemical traits, respectively (Dataset S2). First, we confirmed significant species differences in all traits within each root class using a one‐way ANOVA (*P* < 0.001). To examine the effects of taxonomy and symbiosis on the traits in more detail, we present the results of comparisons by the two‐way ANOVA conducted in each diameter class (Table 3). Across the three root classes, the SRL of the angiosperm species was significantly higher than that of the gymnosperms (Fig. 3a–c; *P* < 0.001, Table 3). Similarly, the RTD of the angiosperm species was significantly higher than that of the gymnosperms (*P* < 0.001) except for those in the 0.5‐mm class (Fig. 3d–f; Table 3). The cortex ratio of the AM species in the 0.5‐mm class was significantly higher than that in the EM species (Fig. 3g; Table 3). In contrast, the stele ratio of the EM species in the 0.5‐mm class was higher than that of the AM species (Fig. 3j; Table 3).

**Fig. 3:**
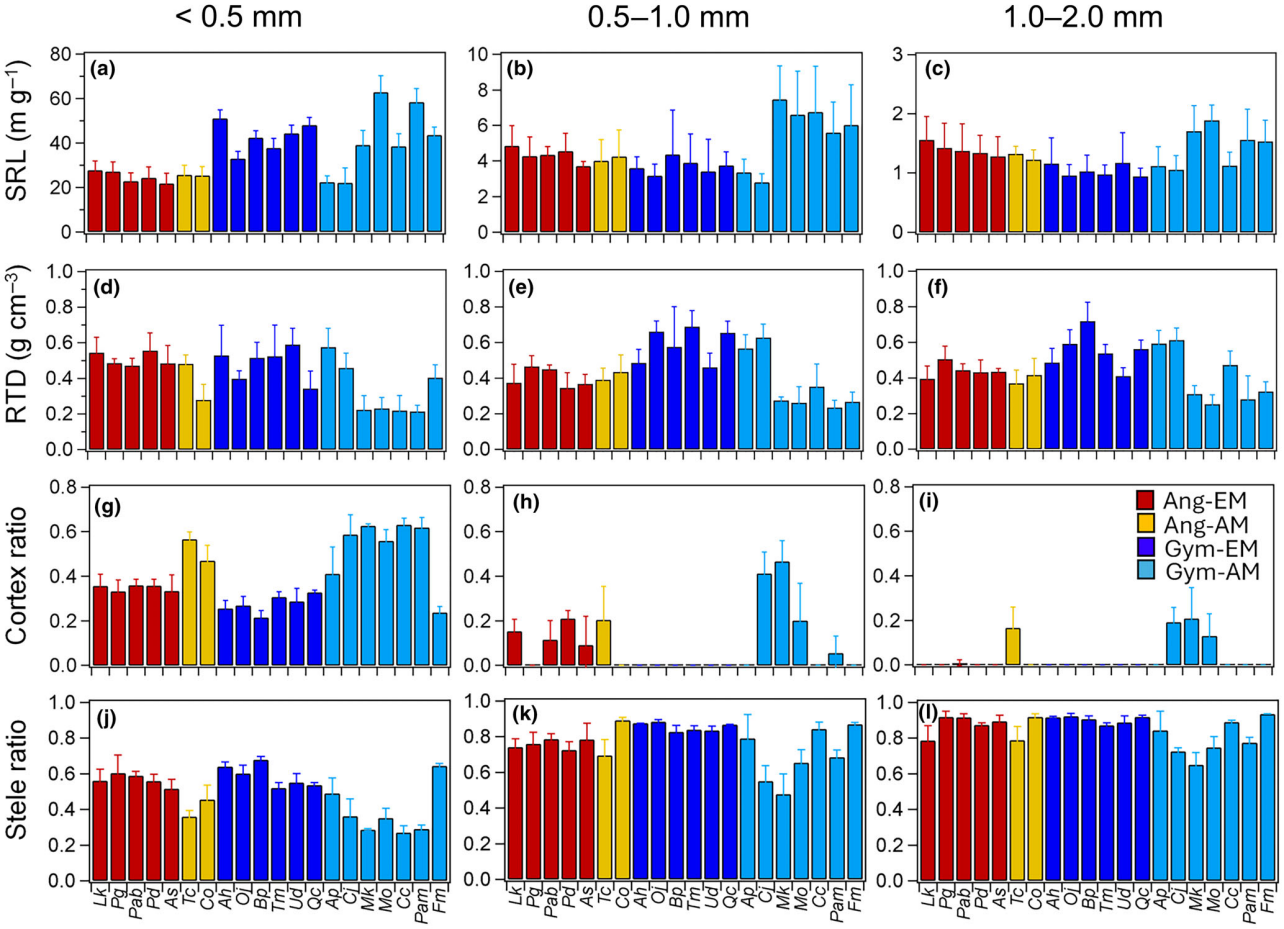
Root morphological traits of three diameter classes in the 20 woody species measured in this study. (a–c) Specific root length (SRL), (d–f) root tissue density (RTD), (g–i) cortex ratio, and (j–l) stele ratio. Error bars represent one SE (*n* = 6). Species abbreviations are shown in Table 1.

**Fig. 4:**
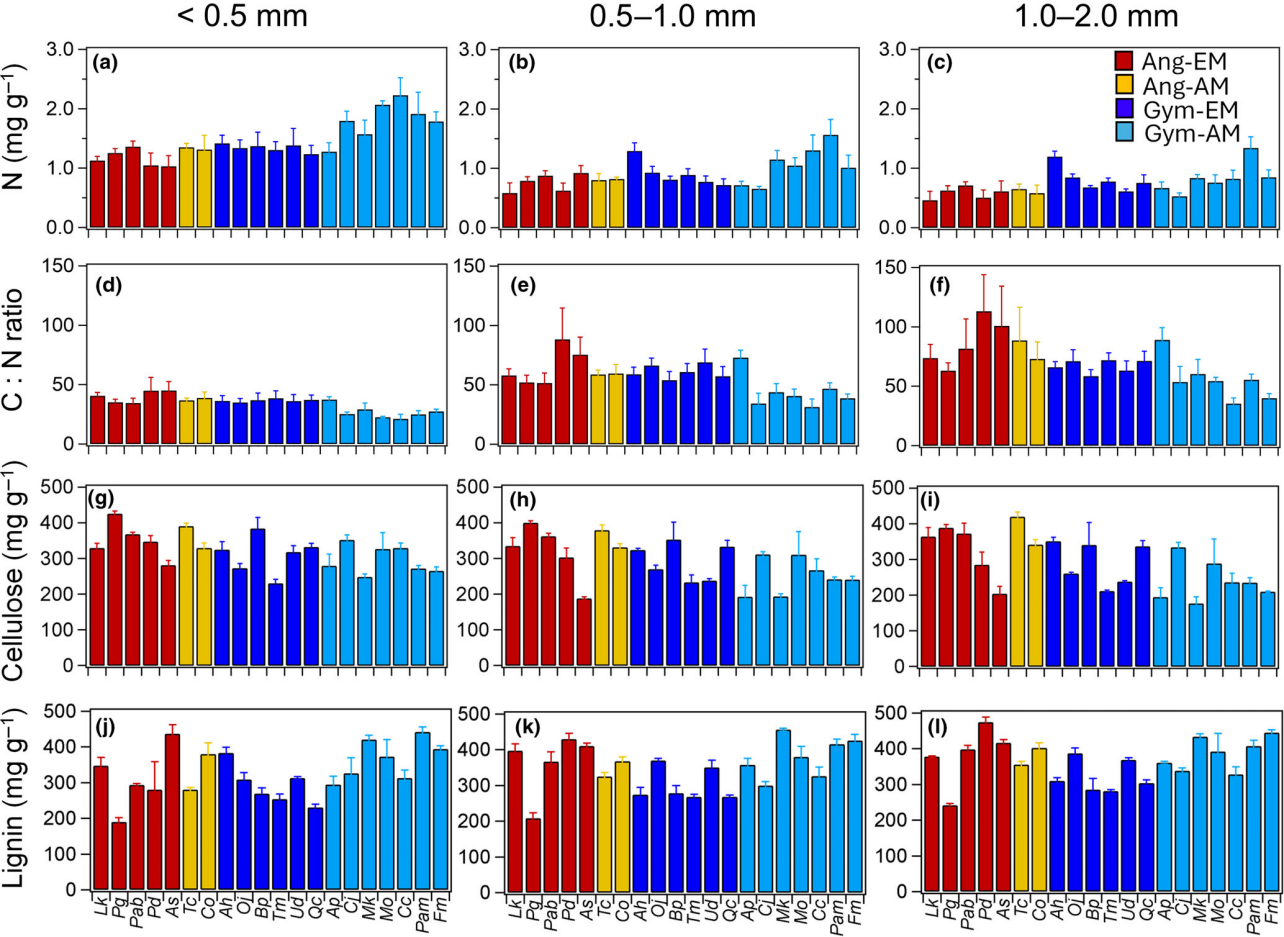
Root chemical traits of three diameter classes in the 20 woody species examined in this study. (a–c) Nitrogen content, (d–f) C:N ratio, (g–i) cellulose content, and (j–l) lignin content. Error bars represent one SE (*n* = 6). Species abbreviations are shown in Table 1.

**Table 3:** Results of two‐way analysis of variance (ANOVA) conducted to test the effect of taxonomy (T), symbiosis (S), and their interaction (T × S) on the root traits.

Trait	Factor	Root diameter class			References
		0–0.5 mm	0.5–1.0 mm	1.0–2.0 mm	
SRL	T	**< 0.001**	0.198	**< 0.001**	Fig. 3(a–c)
	S	0.868	0.765	0.374	
	T × S	0.298	**< 0.01**	**< 0.01**	
RTD	T	0.469	**< 0.001**	**< 0.001**	Fig. 3(d–f)
	S	**< 0.01**	0.801	0.275	
	T × S	0.667	**< 0.001**	0.073	
Cortex ratio	T	**< 0.05**	**< 0.05**	0.942	Fig. 3(g–i)
	S	**< 0.001**	0.863	**< 0.05**	
	T × S	0.134	**< 0.05**	0.749	
Stele ratio	T	0.387	**< 0.05**	0.257	Fig. 3(j–l)
	S	**< 0.01**	0.533	0.501	
	T × S	0.304	**< 0.01**	0.066	
N	T	**0.001**	**< 0.05**	**< 0.001**	Fig. 4(a–c)
	S	0.108	0.553	0.653	
	T × S	0.208	0.305	0.868	
C : N ratio	T	**< 0.01**	**< 0.05**	**< 0.001**	Fig. 4(d–f)
	S	0.344	0.277	0.454	
	T × S	0.059	0.692	0.685	
Cellulose	T	**< 0.001**	**< 0.001**	**< 0.001**	Fig. 4(g–i)
	S	0.058	0.314	0.529	
	T × S	0.175	0.79	0.93	
Lignin	T	0.998	0.181	0.074	Fig. 4(j‐l)
	S	**< 0.001**	0.429	0.643	
	T × S	0.883	**< 0.05**	**< 0.05**	

The analysis was conducted in each root diameter class. The value indicates the *P*‐value, and the significant effect is in bold font.

Across the root classes on the chemical traits, the N and cellulose concentrations of the angiosperms were higher than those of the gymnosperms (*P* < 0.05; Fig. 4a–c; Table 3); in contrast, the CN ratio in angiosperms was lower than that in gymnosperms (Fig. 4d–f; *P* < 0.05). Meanwhile, the lignin concentration in the EM species in the 0.5‐mm class was significantly higher than that in the AM species (Fig. 4j–l; Table 3).

### Prediction of root traits using spectral reflectance

The root trait data for the training and validation datasets appeared similar (Table S1). Table 4 indicates the performance of the PLSR models using three different spectral regions, such as VIS–NIR, SWIR, and all spectral regions. The model's accuracy was tested in both calibration and validation processes. In all tested cases, the estimation error as rRMSE tended to be similar or slightly higher in the validation than in the calibration. When we focus on the effect of used spectral regions on the accuracy, the estimation error of all root traits, except for the cortex ratio, was smaller in the cases using spectral reflectance, including SWIR spectral region (i.e. SWIR and all spectral regions), than in the case using only the VIS–NIR spectral region (Table 4). This trend was observed especially in diameter and SRL, and the rRMSE decreased by *c*. 20% when SWIR spectral reflectance was included in the input data (Table 4). The evaluation of the RPD among the spectral regions was similar to that of the rRMSE. On the other hand, the estimation accuracy differed obviously among the target traits. For example, when we focused on the rRMSE of validation at the use of all spectral regions, rRMSE showed large variations from 14.2% (D) to 73.8% (cortex ratio) (Table 4).

**Table 4:** Results of the calibration and validation of the PLSR models developed for estimating root characteristics.

Spectral region of input data	Trait	Factor	Calibration					Validation				
			*n*	*R* ^2^	RMSE	rRMSE (%)	RPD	*n*	*R* ^2^	RMSE	rRMSE (%)	RPD
VNIR, 458.4–959.6 nm	D (mm)	12	240	0.89	0.18	19.4	2.98	120	0.65	0.35	38.7	1.68
	SRL (m g^−1^)	2	240	0.62	11.1	79.8	1.63	120	0.60	11.4	82.0	1.59
	RTD (g cm^−3^)	3	240	0.35	0.13	28.4	1.24	120	0.38	0.12	27.3	1.28
	Cortex ratio	2	120	0.57	0.14	76.6	1.52	60	0.66	0.12	**71.4**	**1.72**
	Stele ratio	2	120	0.63	0.12	17.1	1.64	60	0.70	0.11	14.7	1.85
	N (mg g^−1^)	2	240	0.43	0.3	31.8	1.33	120	0.45	0.3	30.0	1.36
	C : N ratio	2	240	0.27	20.1	38.0	1.17	120	0.40	17.2	32.0	1.29
	Cellulose (mg g^−1^)	1	120	0.03	70.8	23.6	1.02	60	0.01	70.9	23.8	1.01
	Lignin (mg g^−1^)	2	120	0.26	62.2	18.0	1.16	60	0.29	61.0	17.4	1.19
SWIR, 966.7–2390.8 nm	D (mm)	9	240	0.95	0.12	13.1	4.41	120	0.95	0.13	14.3	4.21
	SRL (m g^−1^)	8	240	0.80	8.0	57.5	2.27	120	0.77	8.8	63.3	2.07
	RTD (g cm^−3^)	7	240	0.54	0.11	23.9	1.48	120	0.47	0.11	**25.3**	**1.38**
	Cortex ratio	4	120	0.57	0.14	**75.9**	**1.54**	60	0.61	0.13	76.0	1.62
	Stele ratio	8	120	0.69	0.11	15.5	1.81	60	0.74	0.11	14.6	1.95
	N (mg g^−1^)	8	240	0.54	0.3	**28.4**	**1.49**	120	0.49	0.3	29.2	1.39
	C : N ratio	8	240	0.50	16.6	**31.5**	**1.42**	120	0.36	17.9	33.2	1.24
	Cellulose (mg g^−1^)	1	120	0.08	69.0	23.0	1.05	60	0.02	71.31	23.9	1.00
	Lignin (mg g^−1^)	8	120	0.49	51.6	**15.0**	**1.40**	60	0.37	57.33	16.4	1.27
Full, 458.4–2390.8 nm	D (mm)	14	240	0.95	0.11	**12.4**	**4.65**	120	0.95	0.13	**14.2**	**4.25**
	SRL (m g^−1^)	12	240	0.83	7.6	**54.3**	**2.40**	120	0.80	8.1	**58.5**	**2.24**
	RTD (g cm^−3^)	9	240	0.58	0.10	**22.8**	**1.55**	120	0.46	0.12	25.6	1.36
	Cortex ratio	1	120	0.56	0.14	77.2	1.51	60	0.63	0.12	73.8	1.66
	Stele ratio	10	120	0.75	0.10	**14.1**	**1.99**	60	0.74	0.10	**13.8**	**1.99**
	N (mg g^−1^)	3	240	0.43	0.3	31.8	1.33	120	0.48	0.3	**29.2**	**1.40**
	C : N ratio	5	240	0.32	19.4	36.7	1.21	120	0.42	16.8	**31.2**	**1.32**
	Cellulose (mg g^−1^)	9	120	0.49	51.4	**17.1**	**1.41**	60	0.34	57.7	**19.4**	**1.24**
	Lignin (mg g^−1^)	4	120	0.36	57.7	16.7	1.26	60	0.42	55.2	**15.8**	**1.33**

The unit of RMSE is the same as each trait. Characters in bold indicate the minimum rRMSE or maximum RPD among three spectral ranges.

Since the model using the full spectral region (VIS–NIR and SWIR) generally provided the highest accuracy across a broad range of morphological and chemical traits, it was selected as the representative model for further trait prediction and spatial visualization. The scatterplots of the model validation outputs for all spectral regions are shown in Fig. 5. The maregression lines between the estimated and observed values were close to 1 : 1 for all traits (Fig. 5; Dataset S2), and all relationships were significant (*P* < 0.001). Taxonomy and symbiosis did not clearly affect these relationships (Fig. 5). A combination of relatively high *r* ^2^ values > 0.7 and small rRMSE values < 20% was observed for D and the stele ratio (Figs. 5a,e). Although the *r* ^2^ value in the SRL was the second highest, the rRMSE was the second highest because the model could not estimate low SRL values < 5 m g^−1^ (Fig. 5b). The corrected SRL in this study showed great variation depending on the diameter classes, and the distribution of SRL at 1‐ to 2‐mm‐diameter class was biased toward particularly low values less than 5 m g^−1^ (Fig. 3c). When PLSR model analysis was performed for logarithmic transformed SRL (log SRL), *r* ^2^ and rRMSE in validation were improved from 0.80 in SRL to 0.89 in log SRL and from 58.5% in SRL to 44.9% in log SRL, respectively (data not shown). On the other hand, a weak estimation capacity in the low‐value range was also observed for the cortex ratio (Fig. 5d). However, the effect of logarithmic transformation did not sufficiently improve the model performance because about half of the cortex ratio was zero (Fig. 3g–i).

**Fig. 5:**
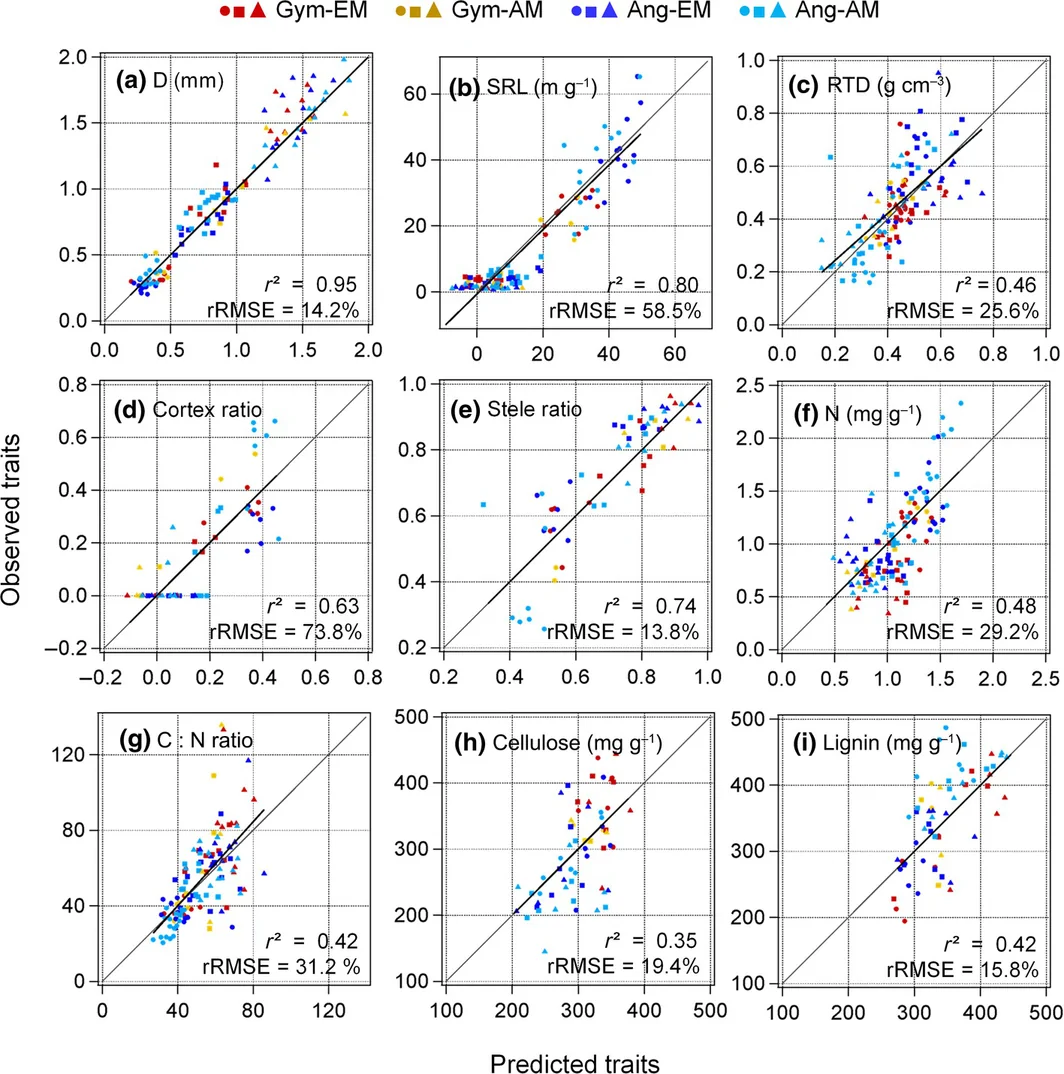
Relationship between the observed and estimated root traits. Panels represent the validation results for (a) root diameter (D), (b) specific root length (SRL), (c) root tissue density (RTD), (d) cortex ratio, (e) stele ratio, (f) nitrogen content, (g) C : N ratio, (h) cellulose content, and (i) lignin content (see Table 4 for the detailed statistical metrics of each model). The estimation values were used by the model at the combined spectral region using visible to near‐infrared region (458–960 nm) and shortwave infrared region (967–2391 nm) and calculated by the partial least squares regression model shown in Table 4. Different symbols indicate root diameter classes: circles, < 0.5 mm; triangles, 0.5–1.0 mm; and squares, 1.0–2.0 mm. Solid and grey lines indicate a regression line passing through the origin and 1 : 1 reference line, respectively.

In this study, to understand the importance and contribution of spectral wavelengths in the regression model, spectral features of VIP and the standardized coefficients were calculated (Burnett *et al*., 2021). The VIP and coefficients of the regression model in the test case using all wavebands (Figs S1, S2). As shown in Fig. S2, the VIP spectral feature showed distinct patterns depending on the type of root trait. All morphological traits except RTD (i.e. D, SRL, cortex, and stele) and two chemical traits, such as N and C : N ratio, showed similar patterns of VIP spectral features. These traits showed one large peak with high VIP values exceeding 0.8 between the wavebands at the VIS region *c*. 627–668 nm and the shorter wavebands at the SWIR region *c*. 1355–1395 nm. This value is considered as a general threshold for assessing the importance of VIP (Wold *et al*., 2001). The small valleys around water absorption bands at 970 and 1200 nm, and one spiky peak near the edge of two spectral imagers (960–967 nm; Table 2), did not have a strong impact that pushed the VIP values below 0.8 (Fig. S2). On the other hand, the remaining three traits, such as RTD, cellulose, and lignin, showed large peaks of high VIP values at a wider spectral range between 578–582 nm and 1569–1575 nm, and three small peaks with high VIP exceeding 0.8 had appeared at SWIR spectral regions *c*. 1685, 1945, and 2310 nm as center wavelength (Fig. S2). As shown in Fig. S2, the spectral features of the coefficients tended to be high frequency with a peak width of several tens of nanometers and were distributed across the entire observed spectral range for root traits with a large number of factors selected in the PLSR model. For the cortex ratio and N, which had fewer selected factors, coefficients with relatively large absolute values were observed in VIS, NIR, and the shorter wavelength bands of the SWIR spectral region, where high VIP was observed.

### PCA results of observed and predicted fine‐root traits

The PCA was performed using four key traits (D, N, SRL, and RTD) to characterize the RES across three diameter classes. For the observed traits, the first two principal components (PC1 and PC2) accounted for 78.3–85.1% of the total variation (Fig. 6a–c; Table S2). PC1 was primarily defined by the trade‐off between acquisitive traits (SRL and N concentration) and structural/conservative traits (D and RTD). This pattern was consistent across all diameter classes, although the separation between angiosperms and gymnosperms was most pronounced in the thinnest root class (< 0.5 mm; Fig. 6a).

**Fig. 6:**
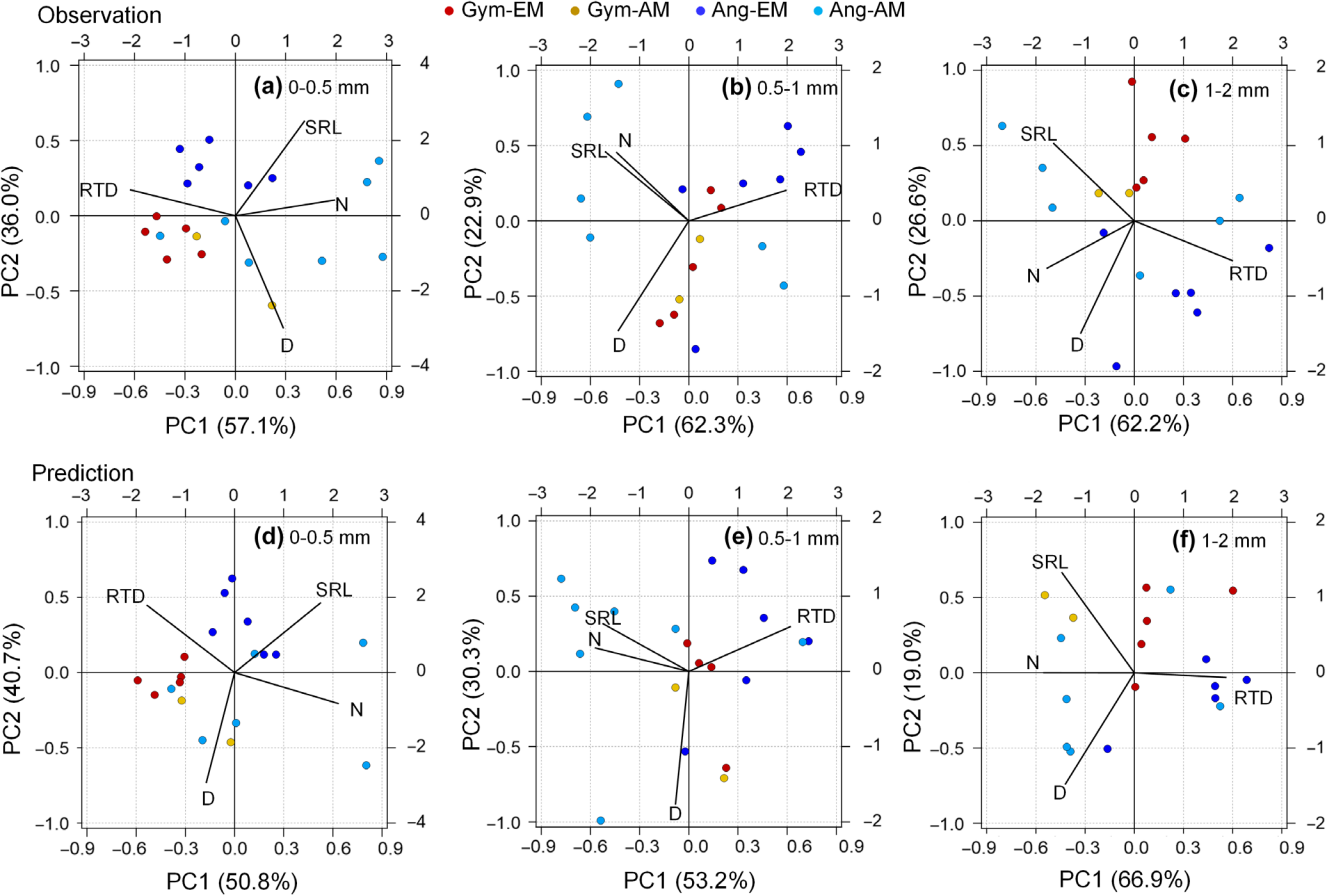
Principal component analysis of root traits of 20 tree species based on (a–c) observation and (d–f) prediction by partial least square regression model. Root sizes were classified into three groups: < 0.5, 0.5–1.0, and 1.0–2.0 mm. Tree species were classified into four groups: gymnosperm (GYM)‐EM, GYM‐AM, angiosperm (ANG)‐EM, and ANG‐AM.

Using traits predicted from imaging spectroscopy, the PCA showed that PC1 and PC2 accounted for 76.5–85.3% of the variation (Fig. 6d–f; Table S2). The PCA distributions of the predicted values were remarkably similar to those of the observed values, with trait vectors and species clustering (e.g. the wider variation in EM roots than AM roots) well‐reproduced in the predicted models. This high degree of consistency between the observation‐based and prediction‐based PCAs demonstrates that our spectral estimation models can effectively capture the multidimensional structure of the RES across different root sizes.

### Visualization of the spatial distribution of root N

Fig. 7 presents the map of the predicted root N concentration in the thinnest root class (< 0.5 mm), calculated using the SWIR reflectance image. Across all 20 woody species, the estimated N concentration exhibited a clear spatial gradient within individual root systems, typically being higher in lower order roots than in higher order roots. Regarding interspecific variation, deciduous angiosperms (ang‐AM), such as *Fraxinus mandshurica* and *Magnolia* species, displayed higher estimated N concentrations compared to gymnosperms (gym‐EM), including *Pinus densiflora*, *L. kaempferi*, and *A. sachalinensis* (Fig. 7).

**Fig. 7:**
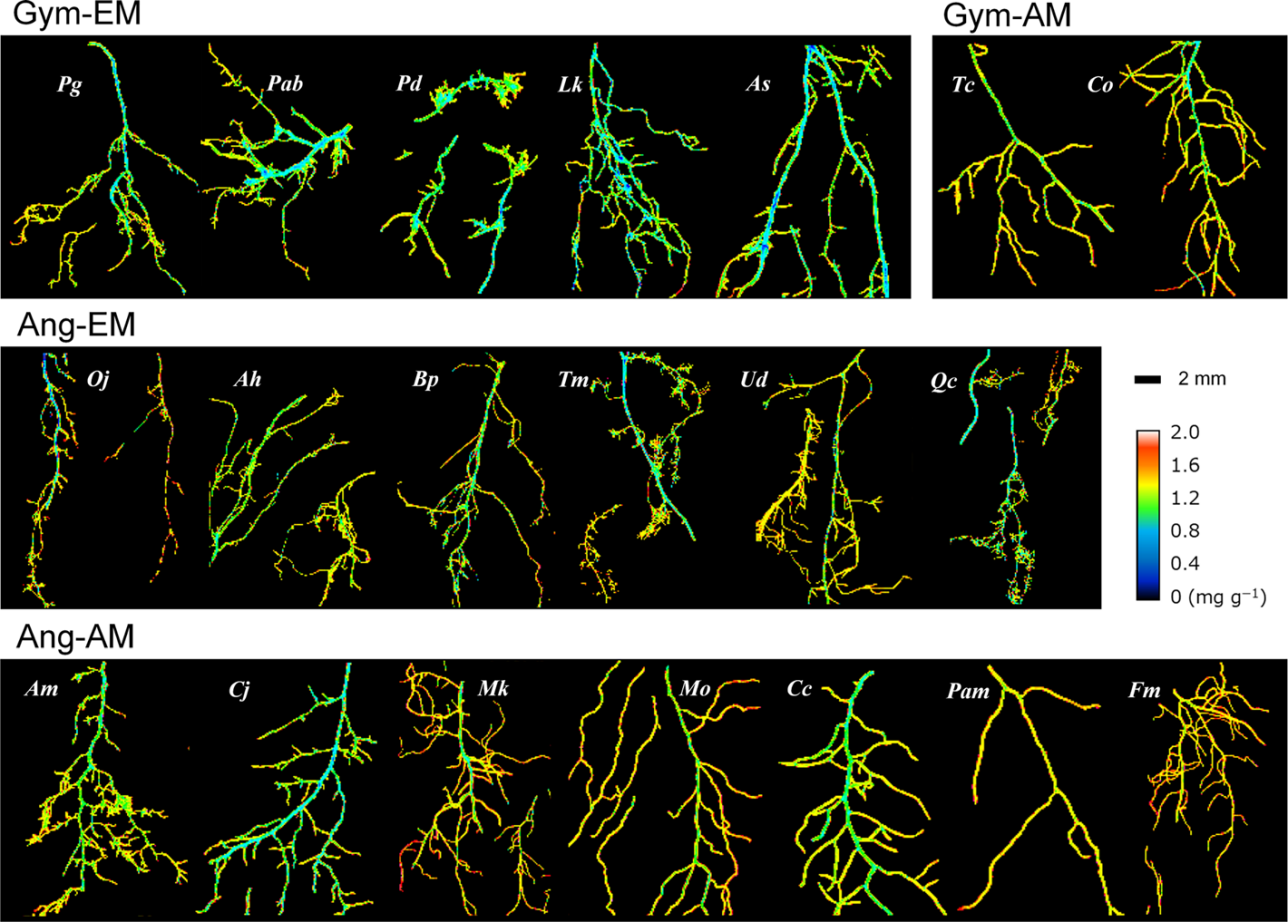
Maps of the estimated root nitrogen concentration in the < 0.5‐mm‐diameter classes of 20 woody species based on the imaging spectroscopy using the shortwave infrared spectral region. The shown image is a part of the root system in each species. Species abbreviations used in the figure panel labels are detailed in Table 1.

## Discussion

This study demonstrates that high‐resolution imaging spectroscopy is a powerful, highly efficient, and reagent‐free tool for predicting multidimensional fine‐root traits in woody species. To our knowledge, this is the first study to quantify the efficacy of spectral reflectance in predicting a comprehensive set of traits from intact fresh root samples across diverse species. Our results highlight that while morphological traits (e.g. diameter and SRL) and N concentration can be robustly predicted, others such as cellulose and lignin were less reliably predicted. Crucially, we show that the trait space derived from these predicted values accurately reproduced the observed multidimensional trait space (Fig. 7), underscoring the high fidelity of our spectroscopic approach. As shown in Fig. 2, the spectral reflectance profiles across the 20 tree species exhibited consistent patterns.

### Spectral reflectance of tree fine roots

In this study, two hyperspectral cameras were used to capture and analyze the spectral reflectance of fresh living fine roots in an indoor setting. Because the root samples were small, we developed a system that uses a low‐reflectance background to minimize secondary reflectance contamination from the background behind the root sample. It is well known that the spectral reflectance features of green leaves in the VIS, NIR, and SWIR regions are associated with pigments, structure, water content, and organic components such as amino acids, lignin, and cellulose (Curran, 1989; Slaton *et al*., 2001; Nakaji *et al*., 2019; Cotrozzi *et al*., 2020; Ji *et al*., 2024). Although reports on the hyperspectral reflectance of fresh fine roots are scarce, VIS reflectance decreased, and NIR reflectance increased as the live fine roots of poplar cuttings aged (Nakaji *et al*., 2008). Tanikawa *et al*. (2019) reported a positive relationship between fine‐root diameter and NIR reflectance in Japanese 12 tree species. Generally, NIR reflectance increases with plant tissue development (i.e. increasing thickness or diameter) due to the thickness‐dependent increase of multiple scattering from plant organs that contain several energy‐absorbing substances in the NIR spectral region (Slaton *et al*., 2001). In this study, NIR reflectance increased with increasing root diameter at the NIR region (700.8–959.6 nm, 64 bands) (Fig. 2). In this study, large interspecific differences were observed respectively in the intensity of VIS reflectance and inclination at the slope from VIS to NIR spectral regions (Fig. 2). Although the mechanisms underlying these differences have not yet been elucidated using pigment analysis, this difference indirectly supports the findings of Tanikawa *et al*. (2019), wherein the brightness, saturation, and hue of fine roots can explain species differences, suggesting that hyperspectral reflectance is a key technique for future species discrimination.

### Root trait estimation using the PLSR model

First of all, we discussed the effective wavelength of the PLSR model in this study. Focusing on the spectroscopic estimation of N and cellulose in plant organs, Serbin *et al*. (2014) conducted a literature review in the case of plant leaves, and they predicted that the SWIR spectral region contains important information on many plant traits such as carbohydrates and proteins. They have constructed PLSR models of leaf N and cellulose collected in cool‐temperate and boreal forests and reported that their VIPs are high in the SWIR region, especially in the longer wavelength bands. While our results show that VIPs are particularly high at NIR and shorter wavelengths of SWIR (Fig. S2), the prediction accuracy was improved by the use of reflectance at the SWIR spectral region (Table 4). A simple comparison between the plant roots and leaves of the wavelength‐dependent VIP is likely to be cautionary due to the large differences in the chemical composition and tissue structure among these plant organs. However, our results suggest that there is at least a common view that SWIR is effective for trait estimation.

Regarding the model performance, we could compare the estimation accuracy with that of previous studies using indices such as RPDs and rRMSE. Although the PLSR model with the spectral reflectance of fresh fine roots is rarely studied, some researchers have attempted using the spectral absorption or reflectance of dry powder samples to estimate root traits. Picon‐Cochard *et al*. (2009) used spectral absorption at the VIS–NIR and SWIR regions and reported a very high estimation accuracy (RPD of 3.37) in N concentration. Elle *et al*. (2019) estimated the lignin concentration in dried powder of herbaceous roots (4 functional types, 60 species) by performing PLSR using VIS‐SWIR absorbance and obtained an RPD of 2.67. These RPDs are higher than the RPDs in the best model (1.40 for N and 1.33 for lignin concentrations) in our study (Table 4). As the effects of H_2_O and structure can be uniformly reduced in dry powder samples, and spectral absorption is more sensitive than reflectance (Bellon‐Maurel & McBratney, 2011), we believe that the lower RPDs obtained in this study than in previous experimental studies are reasonable. However, even in fresh samples, the RPDs in D, SRL, and stele ratio obtained in this study were ≥ 2, which is considered an excellent estimation accuracy in agriculture and food science (Chang *et al*., 2001). Thus, a significant advantage of applying imaging spectroscopy to intact fresh roots is the substantial reduction in time and cost by eliminating labor‐intensive processing, such as drying and grinding. Furthermore, this approach enables the simultaneous estimation of morphological and structural traits (e.g. SRL, RTD, and D) that would otherwise be lost during traditional chemical analysis. The imaging spectroscopy method enabled the identification of spatial distribution even within a single root tip and estimated sequential functional shifts at the targeted points without a cutoff. The relatively high RPDs in D and SRL are attributed to the high correlation between NIR reflectance and organ thickness and between SRL and root diameter (Ma *et al*., 2018). As the pores in the stele are relatively large and are distributed close to the epidermis (Gambetta *et al*., 2013; Endo *et al*., 2021), and the number and size of cellular tissues affect scattering in the NIR spectral region, the stele ratio may be detectable compared with the cortex located in the deeper layer of the root tissue.

For other plant organs, the rRMSE of estimates of N and cellulose in the fresh leaves of tropical forest trees was reported as 12.8 and 14.6%, respectively (Asner & Martin, 2011). The rRMSEs of N, cellulose, and lignin estimates in East Asian tree species were 7.9, 10.4, and 9.1%, respectively (Nakaji *et al*., 2019). In this study, the rRMSEs for N, cellulose, and lignin in the best model were 29.2, 19.4, and 15.8%, respectively (Table 4), indicating that the error tended to be higher for fine roots than for leaves. One possible reason why the prediction of N in roots is less accurate than in leaves may be the absence of Chl. In leaves, N concentration is highly correlated with Chl content, which exhibits distinct spectral signatures in the visible range, making N easier to predict (Serbin *et al*., 2014). In contrast, fine roots lack these pigments, and N prediction must rely on weaker absorption features of N‐H bonds in proteins or amino acids, primarily in the SWIR region. Additionally, the wider range of N concentration within a root system and the complex three‐dimensional architecture of roots may further complicate accurate spectral reflectance measurements. Furthermore, the absorption bands of the ‐NH group at 1450–1480 and 1520–1540 nm were likely masked by the dominant influence of water absorption. It is important to note that the high‐water content in fresh roots exerts a holistic masking effect across the entire reflectance spectrum, reducing the signal‐to‐noise ratio for chemical constituents. This pervasive influence likely accounts for the lower prediction accuracies in our study compared with previous research using dried and ground samples. N concentration is an important root trait for evaluating the functions of roots and the root economic spectrum. The RPD of N concentration was 1.4 (Table 4), which is the threshold for poor accuracy (Picon‐Cochard *et al*., 2009). Thus, the accuracy and applicability of our prediction model could be further enhanced by incorporating spatial nutrient distribution within tissues and integrating three‐dimensional root architecture. Such refinements would allow for a more mechanistic understanding of root absorption function.

### Root traits and the framework of root economics spectrum

Our analysis of the root functional trait space revealed clear coordination among morphological and chemical traits across the 20 woody species. In both observed and predicted datasets, we found that N concentration was negatively correlated with RTD, aligning with the conservation axis of the RES, while SRL and diameter represented the collaboration axis (Fig. 6). Importantly, the PCA space derived from the PLSR‐predicted traits closely mirrored the space constructed from observed measurements. The clustering of species by phylogeny (angiosperms vs gymnosperms) and mycorrhizal associations (AM vs ECM) remained consistent between the two approaches. While certain traits with lower predictive accuracy, such as cellulose, contributed less robustly to the predicted space compared to the observed one, the overall configuration of the RES and the relative positioning of species groups were largely preserved. These results demonstrate that high‐resolution imaging spectroscopy is not only an efficient tool for trait quantification but also a reliable method for ecological synthesis, allowing researchers to reach the same fundamental conclusions about belowground strategies as traditional, labor‐intensive direct measurements.

It should also be noted that some predictions were successful, while others were not. For example, the traits such as diameter and N, which have absolute values, are easier to predict by imaging spectroscopy method. Conversely, traits such as SRL and cortical thickness, which reflect relative values and specific internal structures, are more challenging to predict. Additional research with appropriate replicates for various measurement parameters is needed to establish this methodology.

From the PCA, the first two axes encompassed a plane with a cumulative explanatory power of > 75% of all root trait variation (Fig. 6). The primary axis (PC1) was positively correlated with root diameter, C : N, and stele and negatively correlated with SRL, nitrogen, and cortex ratio, suggesting a trade‐off between resource acquisition and transportation with increasing root diameter, which was similar to the dominant gradient observed in previous studies (Kong *et al*., 2017; Ma *et al*., 2018). The secondary axis was related to lignin and cellulose in relation to changes in RTD, suggesting root mechanical strength and root conservation. The structures of lignin and cellulose facilitate water availability and mechanical strength in tree species (Boerjan *et al*., 2003; Zhang *et al*., 2014; Faust *et al*., 2018). Two distinct and largely independent gradients along the diameters with microbial symbiosis effects, especially angiosperm species, were apparent through PCA. As the EM species tended to have higher scores on the secondary axis than the AM species, we suggest that AM species, especially angiosperms, would exhibit specific strategies that differ from EM species by enhancing cellular activities, nutrient absorption, and transport instead of increasing root length and surface area. Currently, there is a general lack of understanding of how the trait associates trade‐offs with species and functional types in RES, how such trade‐offs affect the physiological processes for plant growth and development along the fast‐slow conservation gradient of economic investment (Weemstra *et al*., 2016; Weigelt *et al*., 2021, 2023), and how these are modulated by environmental conditions (Ostonen *et al*., 2011; McCormack *et al*., 2014; McCormack *et al*., 2015). Our findings indicate that species associating with ECM or AM have different fine‐root traits for developing structure, chemical content, and/or anatomical features necessary for nutrient and water acquisition (Figs 3, 4). This divergence in root traits between mycorrhizal types is supported by Sanaei *et al*. (2026), who demonstrated that the coordination between above‐ and belowground traits can vary across different phylogenetic groups and mycorrhizal associations.

Interestingly, the multidimensional root trait characteristics of the measured data were quite similar to those from the estimated data. This key finding demonstrates the usefulness of the spectral imaging technique for detecting root traits. In addition, this technique can advance our understanding of root traits and resource strategies by efficiently measuring and spatial mapping of root traits across root classes. In recent studies, there has been growing evidence showing multiple dimensions of fine root traits, such as RES, improving our understanding of the root resource acquisition and conservation functions with high precision. However, nowadays, many analyses of existing root traits have been hindered by the difficulty of labor‐intensive manual work based on researchers' experience. Our method has discovered the possibilities to overcome this issue by providing a highly efficient and reagent‐free approach without the need for labor‐intensive chemical digestion or sample grinding. This is especially true for predicting root traits of the fresh samples related to morphology, chemistry, and anatomy of living roots with high precision using imaging spectroscopy. Thus, the imaging spectroscopic techniques employed in this study can be used to estimate multiple characteristic datasets from a single image. Our findings highlight an improved method for nondestructively assessing the multifaceted traits of living roots while maintaining their natural state. In addition, data from spectral analysis using certain measurement conditions, as this time, would have high objectivity and reproducibility, leading to consistent quality compared to manual analysis. The consistency between the observed and predicted PCA results (Fig. 6) confirms that our spectral imaging method not only predicts individual trait values but also preserves the fundamental coordination and trade‐offs among traits, which define the root economics spectrum. Furthermore, the high‐resolution imaging spectroscopy used in this study has the potential to be applied to tree species identification and to quantifying mycorrhizal colonization in future research. These capabilities are essential for shifting from the traditional root economic spectrum to a multidimensional ‘RES’ that includes mycorrhizal collaboration strategies. Integrating our imaging method with advanced machine learning will facilitate a more comprehensive understanding of complex belowground functional strategies in forest ecosystems.

### Practical applications of root imaging spectroscopy to whole root systems

The nitrogen maps generated in this study (Fig. 7) illustrate the unique advantage of imaging spectroscopy over traditional chemical analysis. While conventional methods require milling and pooling samples – thereby masking intraroot heterogeneity – our approach preserves the spatial distribution of traits within intact root systems. This capability opens new avenues for root trait ecology; for instance, it allows researchers to monitor localized physiological responses to nutrient patches in soil or to track temporal changes in root senescence while maintaining sample integrity. By integrating these high‐throughput spectral data, future studies can more accurately characterize the multidimensional RES across diverse biomes and environmental gradients.

### Conclusion and future perspectives

This study demonstrates that imaging spectroscopy with dual hyperspectral cameras is a powerful tool for the objective assessment of fresh fine‐root traits. Our approach captured functional trait variation across diverse species, mycorrhizal groups, and phylogenetic lineages. Notably, the pixel‐based analysis enables ultra‐small‐scale, continuous trait mapping without physical root segmentation. Critically, it also enables high‐resolution mapping of traits across the entire root system without physical segmentation. This capability allows for the quantification of intra‐individual trait heterogeneity, providing a new perspective on how trees fine‐tune their resource acquisition strategies in response to localized soil conditions. Such spatially explicit data will significantly enhance our understanding of root–rhizosphere interactions and the functional complexity of belowground ecosystems.

To further advance belowground research, building comprehensive databases that link spectral reflectance with functional traits across various species and root ages is essential. Despite the challenges of accounting for developmental stages, the rapid and multidimensional information provided by imaging spectroscopy offers a breakthrough for root‐trait studies. Overall, this methodology enhances our ability to characterize fine‐root systems and provides a reliable framework for future forest ecological research.

## Competing interests

None declared.

## Author contributions

NM, NT and TN designed the study, collected the data and analyzed the data. NM and TN drafted the initial version of the manuscript, and all authors provided feedback on earlier drafts. Each author has reviewed and approved the final version of the manuscript.

## Disclaimer

The New Phytologist Foundation remains neutral with regard to jurisdictional claims in maps and in any institutional affiliations.

## Supporting information


**Dataset S1** Spectral reflectance features of fresh roots of 20 tree species in three root classes.
**Dataset S2** Fine‐root traits of observation and prediction of 20 tree species in three root classes.

**Fig. S1** Thresholds for the separation of image pixels into the parts of fine roots and the background plate.
**Fig. S2** Spectral features of variable influence on projection values of the partial least square regression models using the reflectance of all spectral regions.
**Table S1** Root traits of the training data set and the validation data set.
**Table S2** The loading scores from the principal component analysis illustrate the relative contribution of each feature to the resulting principal component based on observation and estimation by partial least square regression model.
Please note: Wiley is not responsible for the content or functionality of any Supporting Information supplied by the authors. Any queries (other than missing material) should be directed to the *New Phytologist* Central Office.

## Data Availability

The data that support the findings of this study are available in Dataset S1 and S2.
