# Supplementary material for: A novel method based on high‐resolution imaging spectroscopy to predict fine‐root traits and the economics space of fresh tree roots
Source: New Phytol. 2026 Jun 23;252(5):1816–31. doi: 10.1111/nph.71366 (PMC13738673; doi:10.1111/nph.71366)
Supplement: Supplementary file 1 [file NPH-252-1816-s001.pdf]

**New Phytologist Supporting Information**

**Article title:** A novel method based on high-resolution imaging spectroscopy to predict fine-root traits and the economics space of fresh tree roots

**Authors:** Naoki Makita, Natsuko Tanikawa, Tatsuro Nakaji

**Article acceptance date: 23 April 2026**

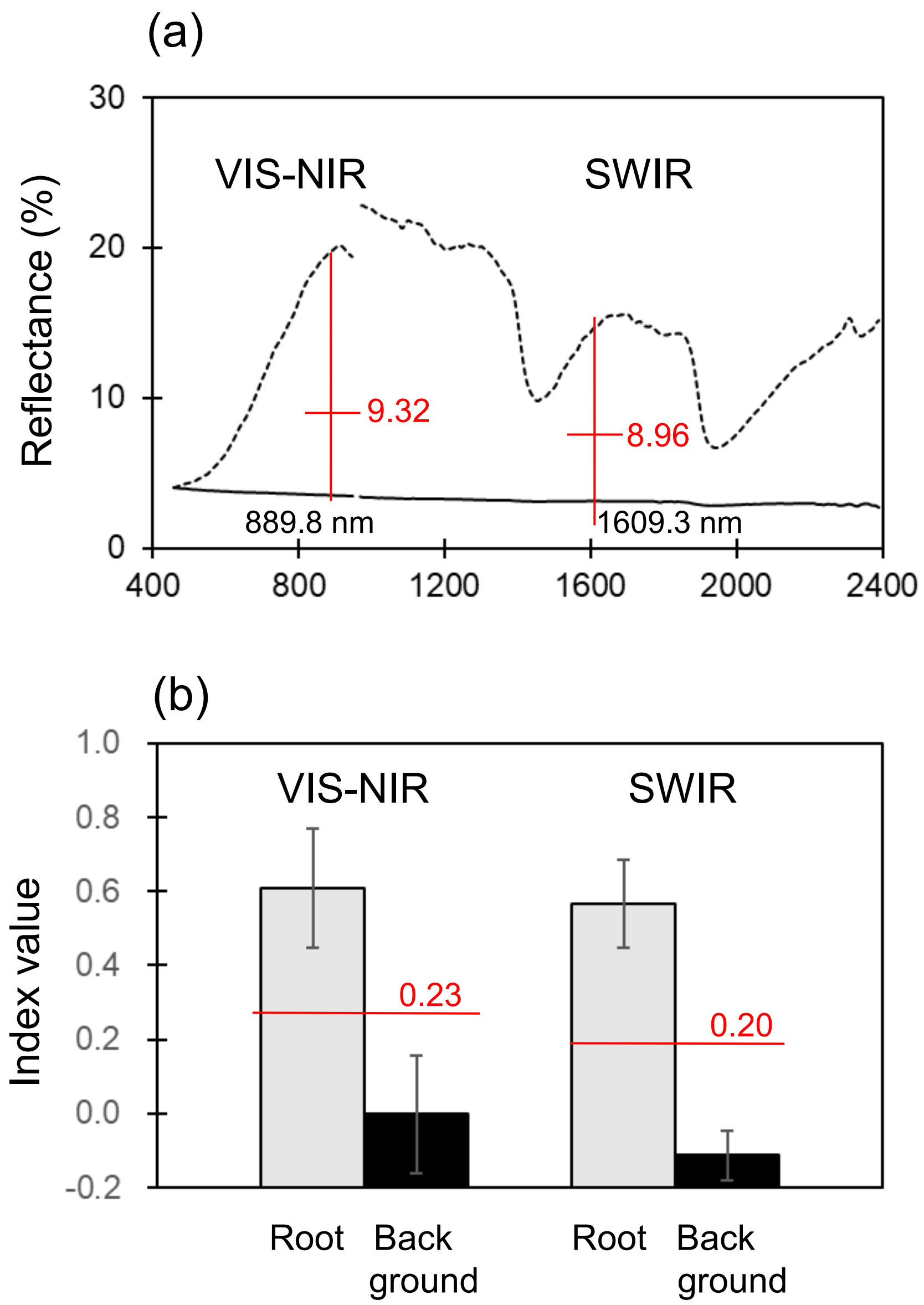


**Figure S1.** Thresholds for the separation of image pixels into the parts of fine roots and the background plate (black cloth). Thresholds of (a) spectral reflectance and (b) normalized difference were selected based on the F value in the comparison between the fine roots and background. In both methods, the difference between the fine root test data and the background was significant (ANOVA, $p < 0.001$, $n = 432$, 20 species).

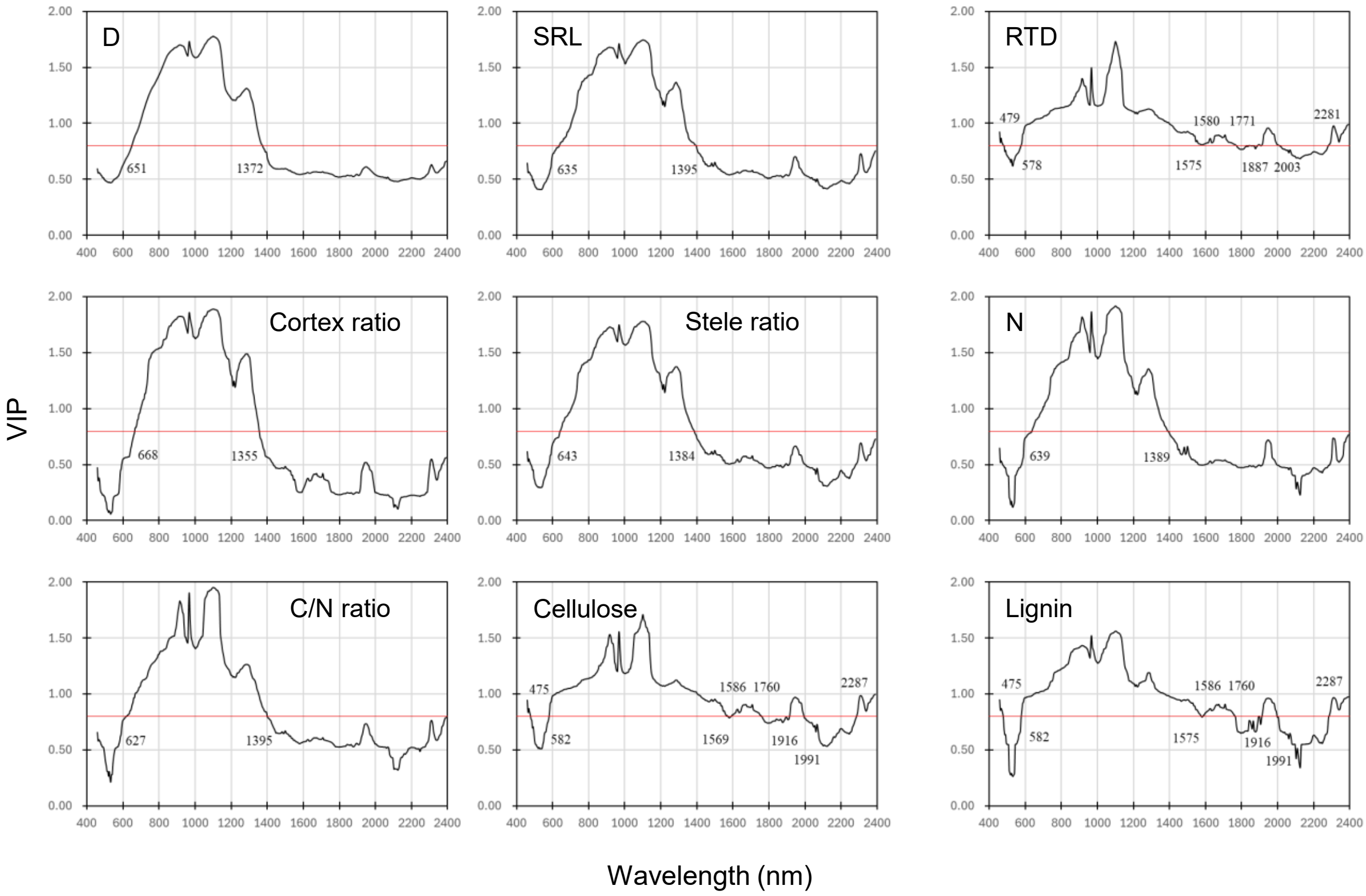


**Figure S2** Spectral features of variable influence on projection (VIP) values of the PLSR models using the reflectance of all spectral regions. The red-colored line indicates the 0.8 of VIP, which is generally considered a highly important variable in the PLSR model. The value in the figure shows the wavelength at the point where the VIP curves and the horizontal line of VIP = 0.8 intersect.

**Table S1.** Root traits of the training data set and the validation data set. The statistical parameters (average, minimum, maximum, and standard deviation) were calculated for the merged samples, including all tree species and all root diameter classes. There were no significant differences among the datasets in all traits.

| | | Morphological trait | | | | | Chemical trait | | | |
|---|---|---|---|---|---|---|---|---|---|---|
| | | D (mm) | SRL (m/g) | RTD ($g/cm^3$) | Cortex ratio | Steal ratio | N (%) | C/N ratio | Cellulose (mg/g) | Lignin (mg/g) |
| Training data | Avg | 0.92 | 13.9 | 0.44 | 0.18 | 0.70 | 10.5 | 52.8 | 300.6 | 345.1 |
| | Min | 0.17 | 0.6 | 0.11 | 0.00 | 0.23 | 2.5 | 17.9 | 130.0 | 170.0 |
| | Max | 2.17 | 91.8 | 0.98 | 0.68 | 0.94 | 25.8 | 186.7 | 437.1 | 498.9 |
| | SD | 0.53 | 18.1 | 0.16 | 0.21 | 0.20 | 4.4 | 23.5 | 72.4 | 72.5 |
| Varidation data | Avg | 0.91 | 13.9 | 0.45 | 0.17 | 0.73 | 10.2 | 53.9 | 298.1 | 350.0 |
| | Min | 0.20 | 0.7 | 0.16 | 0.00 | 0.26 | 3.4 | 20.4 | 144.7 | 194.3 |
| | Max | 2.01 | 89.3 | 0.95 | 0.66 | 0.96 | 23.3 | 135.6 | 442.8 | 487.0 |
| | SD | 0.54 | 18.0 | 0.16 | 0.20 | 0.20 | 4.1 | 22.1 | 71.6 | 72.7 |

**Table S2** The loading scores from the principal component analysis (PCA) analysis illustrate the relative contribution of each feature to the resulting principal component based on observation and estimation by PLSR model, where each row in the table represents root traits and the columns indicate the principal components (PC1, PC2). The illustration of PCA is shown in Figure 6.

| | Observation | | Prediction | |
|---|---|---|---|---|
| | PC1 | PC2 | PC1 | PC2 |
| 0-0.5 mm | | | | |
| SRL | 0.414 | 0.630 | 0.527 | 0.465 |
| RTD | -0.627 | 0.172 | -0.536 | -0.449 |
| D | 0.286 | -0.748 | -0.172 | -0.733 |
| N | 0.594 | 0.104 | 0.635 | 0.206 |
| 0.5-1.0 mm | | | | |
| SRL | -0.511 | 0.461 | -0.573 | 0.156 |
| RTD | 0.596 | 0.203 | 0.622 | 0.298 |
| D | -0.432 | -0.732 | -0.081 | -0.885 |
| N | -0.442 | -0.456 | -0.526 | 0.318 |
| 1.0-2.0 mm | | | | |
| SRL | -0.494 | 0.516 | -0.443 | 0.668 |
| RTD | 0.601 | -0.265 | 0.562 | -0.030 |
| D | -0.53 | -0.316 | -0.422 | -0.743 |
| N | -0.328 | -0.749 | -0.555 | 0.001 |
